# Supplementary figures and images for: TANK prevents IFN-dependent fatal diffuse alveolar hemorrhage by suppressing DNA-cGAS aggregation
Source: Life Sci Alliance. 2021 Nov 24;5(2):e202101067. doi: 10.26508/lsa.202101067 (PMC8616552; doi:10.26508/lsa.202101067)

# Source Data for Figure 6C

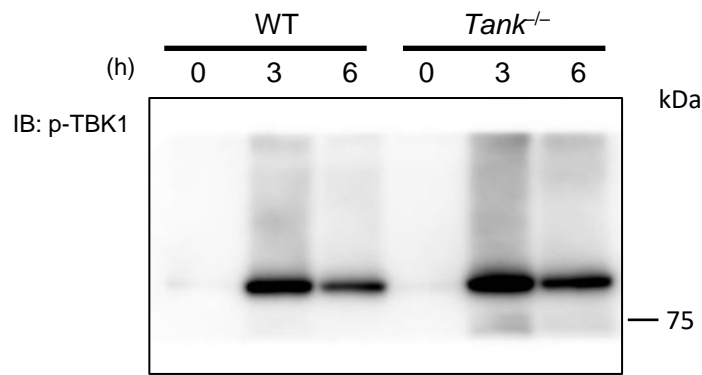

p-TBK1/Actin      1.0    22.9    4.4    0.5    43.3    8.5

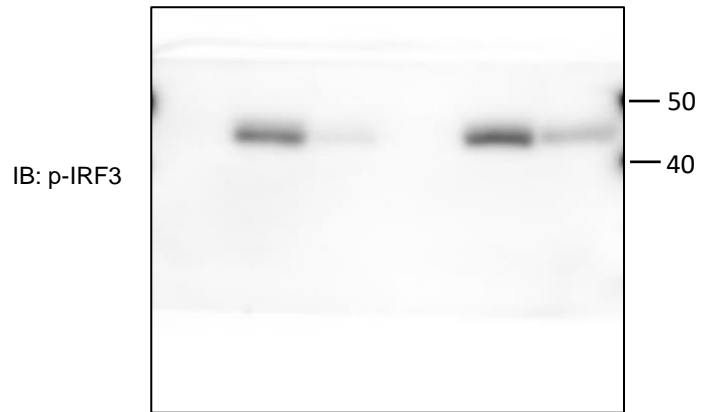

p-IRF3/Actin      1.0    29.8    4.4    0.5    43.2    8.6

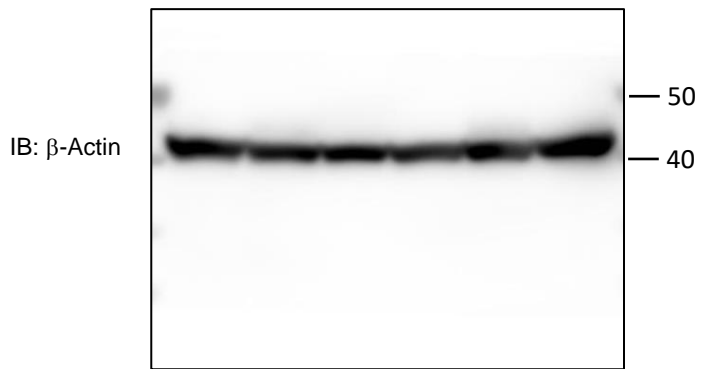

Supplement: Supplementary file 7 [file LSA-2021-01067_SdataF6.2.pdf]

Source Data for Figure 7B

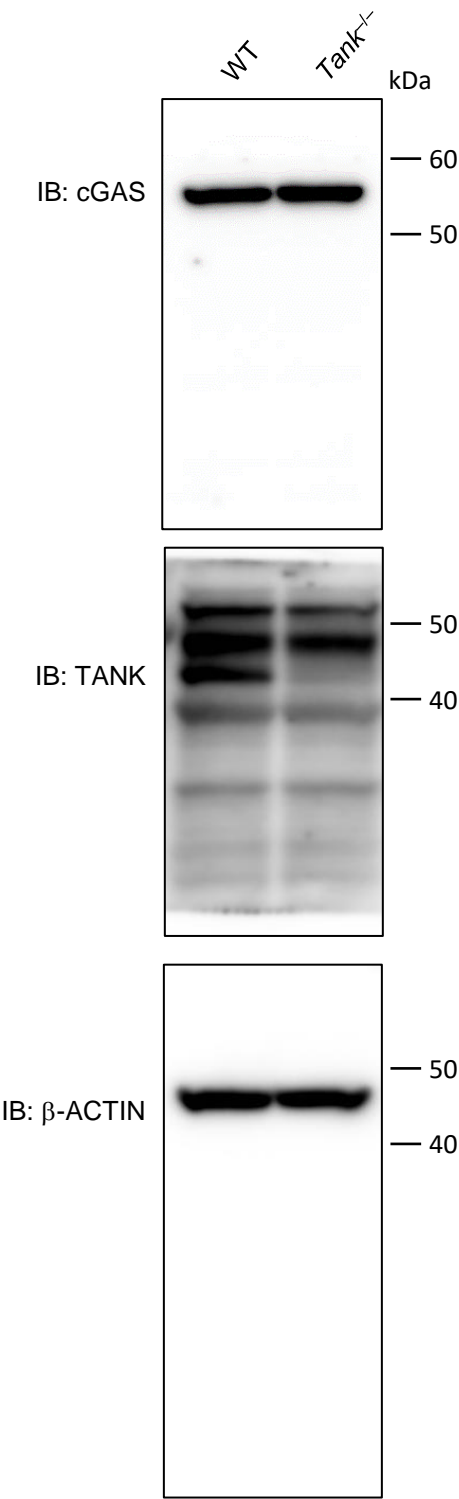

Supplement: Supplementary file 9 [file LSA-2021-01067_SdataF7.2.pdf]
